# Comparative Quality Analysis of Bullous Pemphigoid Information Across Six Social Media Platforms

**DOI:** 10.7759/cureus.81163

**Published:** 2025-03-25

**Authors:** Ezdean Alkurdi, Robert Li, Dany Alkurdi, Omar Alani, Prem Patel, Zachary A Schwager

**Affiliations:** 1 Dermatology, University of Massachusetts Chan Medical School, Worcester, USA; 2 Dermatology, Icahn School of Medicine at Mount Sinai, New York, USA; 3 Dermatology, Lahey Hospital and Medical Center, Burlington, USA

**Keywords:** bullous pemphigoid, facebook, instagram, online medical information, social media, thread, tiktok, twitter, x, youtube

## Abstract

Social media has changed the digital health landscape, altering how the public consumes medical information and sparking concerns about the reliability of medical information. This study aims to assess the quality of online health information related to bullous pemphigoid by evaluating content type, information reliability, and user engagement across different social media platforms, using the Quality Evaluation Scoring Tool (QUEST) and Discerning the Quality of Written Consumer Health Information (DISCERN) questionnaires, quantitative tools designed to measure health information quality. Among the six social media platforms analyzed, educational content was most prevalent on YouTube (Google LLC, Mountain View, California, United States) (90.0%, n=10) and Threads (Meta Platforms, Inc., Menlo Park, California, United States) (100.0%, n=3), while TikTok (ByteDance Ltd., Beijing, China) (n=20) featured 60.0% educational and 30.0% patient experience content. X (X Corp., Bastrop, Texas, United States) (n=18) demonstrated the highest reliability, with a mean QUEST score of 16.8 (SD: 4.5) and a mean DISCERN score of 28.9 (SD: 6.7). In contrast, TikTok had the lowest reliability, with a mean QUEST score of 9.0 (SD: 3.0) and a mean DISCERN score of 20.1 (SD: 3.0). Dunn’s test with Bonferroni correction revealed that TikTok scored significantly lower than X in both QUEST (Mean Difference: -7.83, p < 0.0001) and DISCERN scores (Mean Difference: -8.83, p <0.001). Instagram (n=9) outperformed TikTok in QUEST scores (Mean Difference: 6.66, p <0.001. These findings indicate that X offers the most reliable health information, whereas TikTok consistently ranks lower in reliability.

## Introduction

The advent of new information-sharing technologies, particularly social media, has transformed the way society interacts with medical and health-related information. As of 2023, social media platforms boasted approximately 5.04 billion users worldwide, and approximately 58.5% of adults in the United States reported using the internet to seek medical and health information [1]. This growing reliance on digital resources for health education and decision-making raises critical public health concerns about the quality and validity of shared information. Healthcare providers must remain aware of the content patients may encounter online and can leverage social media to disseminate accurate, evidence-based health information-particularly for rare diseases lacking comprehensive digital resources [2,3].

Bullous pemphigoid (BP) is an autoimmune blistering condition that affects the skin and mucous membranes. Although relatively rare, BP can have a profound impact on patients’ physical and psychological well-being, often causing painful or pruritic blisters, long diagnostic delays, and significant anxiety or distress related to its chronic nature [4,5]. Fewer vetted online resources for BP may compound these challenges and contribute to misinformation and delays in diagnosis.

Social media platforms such as Threads (Meta Platforms, Inc., Menlo Park, California, United States) and X (formerly Twitter) (X Corp., Bastrop, Texas, United States) offer opportunities for BP patients to access health information, participate in digital communities, and connect with advocacy groups. This study aims to quantify and characterize the nature and validity of social media posts related to BP on top social media platforms, specifically by quantifying content quality, with the goal of enhancing our understanding of both the potential benefits and risks of online health information sharing.

## Materials and methods

This study was conducted at the University of Massachusetts Medical School, Worcester, Massachusetts, United States. To examine the social media landscape on BP, we adopted a systematic approach across six of the most popular social media platforms: Facebook (Meta Platforms, Inc.), Instagram (Meta Platforms, Inc.), X, YouTube (Google LLC, Mountain View, California, United States), TikTok (ByteDance Ltd., Beijing, China), and Threads. These platforms were selected due to their extensive user bases and diverse content formats, including text, images, and videos. The primary term searched in this exploration was "bullous pemphigoid," and searches were conducted using new accounts to control for algorithmic biases. This methodology aimed to provide a snapshot of the most recent and popular content related to BP. Each social media platform was searched using specific terms or hashtags related to BP, and top posts were analyzed according to prespecified criteria; additional metrics about each social media source were collected based on creator and content types.

Inclusion and exclusion criteria

The analyzed sources included English-language content relevant to human cases of BP and pertinent to patients, healthcare providers, and the general public. Non-human-related BP content (e.g. about BP in animals) and posts in languages other than English were excluded.

Content quality assessment

To assess content quality, two established quantitative scoring tools: the Quality Evaluation Scoring Tool (QUEST) and Discerning the Quality of Written Consumer Health Information (DISCERN) questionnaires were utilized. QUEST is a questionnaire that assesses various health information quality criteria, including authorship, attribution, conflict of interest, currency, complementarity, and tone [6]. DISCERN is a questionnaire that provides a reliable and valid set of criteria to evaluate online health information [7]. Section 1 of the questionnaire consists of eight questions, which evaluate the reliability of the information; section 2 of DISCERN, which focuses on broader aspects of health information beyond treatment options, was excluded because our aim was to assess the general accuracy and reliability of BP information, rather than specific treatment discussions; QUEST complements DISCERN by evaluating broader content validity, thus mitigating the need for DISCERN Section 2 [7]. Two independent reviewers (EA and RL) evaluated the media and scored them based on the QUEST and DISCERN criteria, completing all data collection in one day.

Data collection and scoring

A comprehensive data set was compiled, which included scores for different metrics of user engagement for each social media post, such as authorship, attribution, conflict of interest (COI), currency, and complementarity for each platform. These metrics were aggregated to form each social media platform's total QUEST and DISCERN scores. 

Statistical analysis

Cohen's weighted kappa was utilized to assess the inter-reliability of QUEST and DISCERN scores assigned by raters. Additionally, given the continuous, non-parametric nature of the QUEST and DISCERN scores, the Kruskal-Wallis test was used to analyze differences between each social media platform, followed by Dunn’s test with an applied Bonferroni correction to determine which specific social media platforms differed from one another. Statistical significance was set at p<0.05. All statistical analyses were performed in R version 4.3.3 (R Foundation for Statistical Computing, Vienna, Austria).

## Results

**Figure 1:**
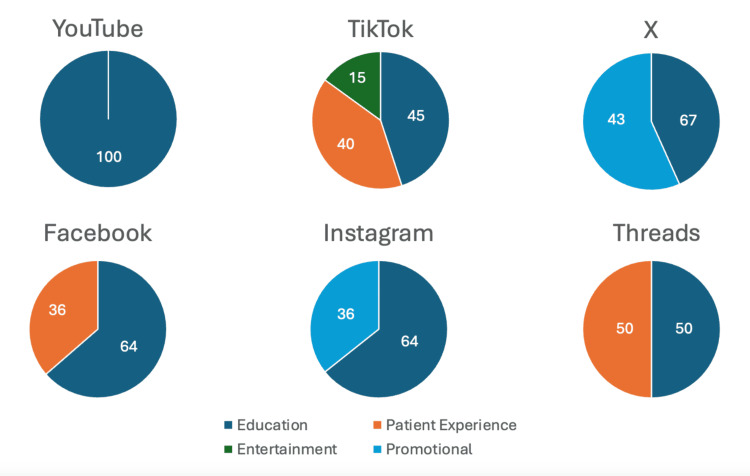
Overview of social media content types for bullous pemphigoid

Analysis of the six social media platforms revealed a varying prevalence of creator types for BP across different channels. Educational content was primarily found on YouTube (90.0%) and Threads (100.0%), while TikTok featured a substantial amount of both educational (60.0%) and patient experience (30.0%) material. Content on X predominantly came from physicians (55.6%) and organizations (44.4%), whereas Facebook was mainly populated by organizations (85.7%) and support groups (14.3%). Instagram largely featured content from organizations (55.6%) and physicians (33.3%). Entertainment content was minimal, present only on TikTok (10.0%), and personal accounts and patient experience content had limited representation across all platforms (Figure 1).

**Figure 2:**
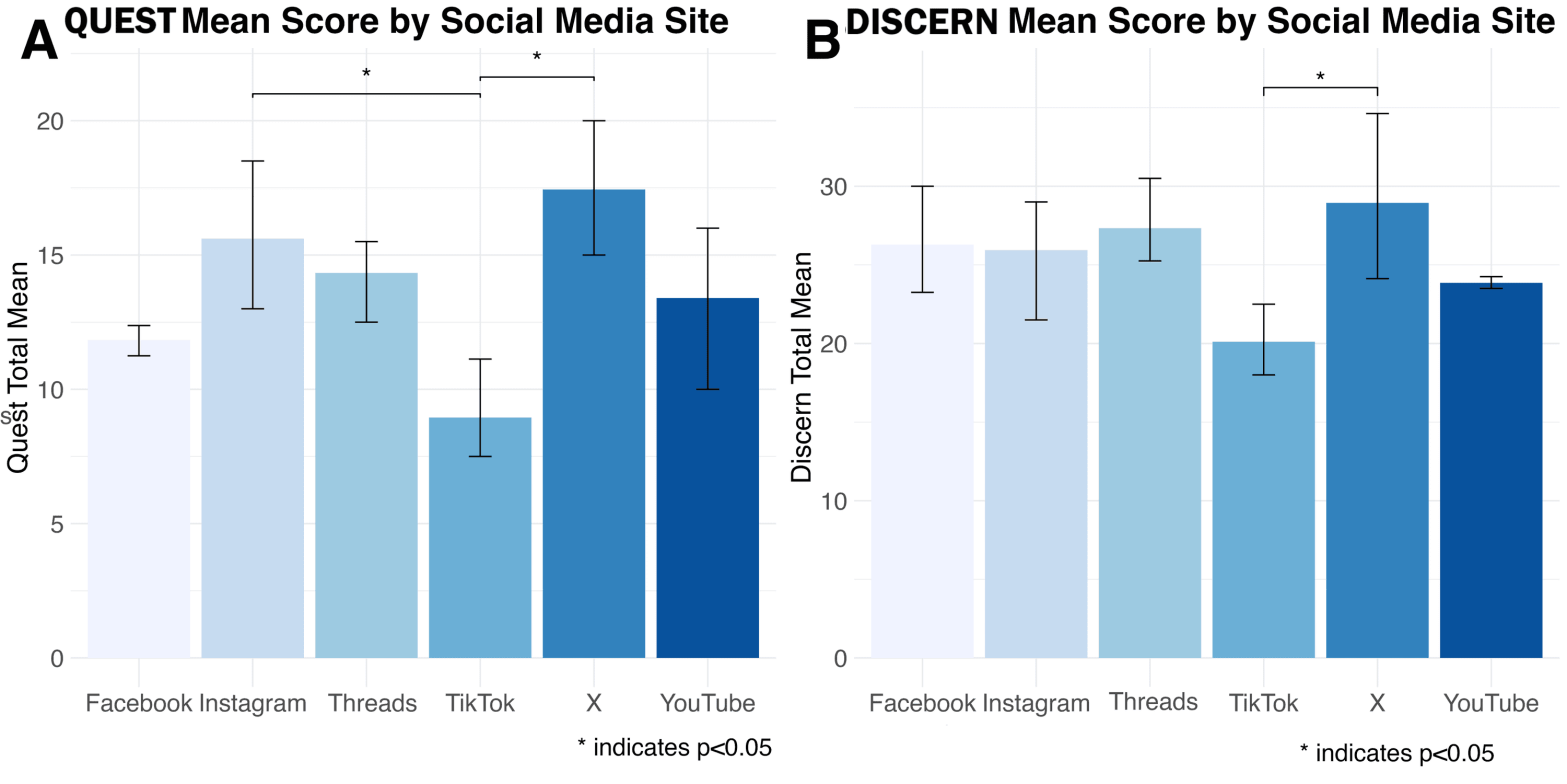
Comparison of QUEST (A) and DISCERN (B) mean scores by social media site QUEST: Quality Evaluation Scoring Tool; DISCERN: Discerning the Quality of Written Consumer Health Information

Inter-rater reliability across all social media sites demonstrated substantial agreement, with substantial agreement among the raters for QUEST scores (Weighted Kappa = 0.742, p < 0.001) and DISCERN questions (Weighted Kappa = 0.569, p < 0.001) (Table 1). The Kruskal-Wallis rank sum test revealed significant differences in QUEST scores across the social media platforms (p<0.001). The scores ranged widely, with X showing the highest score (mean = 16.8, SD = 4.5) and TikTok the lowest (mean = 9.0, SD = 3.0). Similarly, DISCERN scores also exhibited significant differences (p=0.015), although the range was narrower compared to QUEST scores. Again, X had the highest DISCERN score (mean = 28.9, SD = 6.7) while TikTok scored the lowest (mean = 20.1, SD = 3.0). This indicates that the reliability of the health information provided by TikTok rates consistency lower than all other platforms, and that X provides the most reliable source of information (Table 1). Figure 2 depicts the average QUEST and DISCERN scores exhibited by each platform.

**Table 1:** Comparison of QUEST and DISCERN scores by social media platform and respective inter-rater reliability Mean QUEST and DISCERN scores were calculated along with standard deviation for each social media platform. Kruskal-Wallis Rank Sum Test was used to determine any differences between platforms. Reviewer scores for both QUEST and DISCERN were analyzed using Cohen’s weighted kappa test. Values closer to 1 indicate better inter-rater reliability. *Denotes Statistical Significance (p < 0.05); ^2^Kruskal-Wallis Rank Sum Test QUEST: Quality Evaluation Scoring Tool; DISCERN: Discerning the Quality of Written Consumer Health Information

^Category^	Facebook (N = 7), mean (SD)	Instagram (N = 9), mean (SD)	Threads (N = 3), mean (SD)	TikTok (N = 20), mean (SD)	X (N = 18), mean (SD)	YouTube (N = 10), mean (SD)	p-value^2^
QUEST	12.9 (3.2)	15.6 (3.5)	14.3 (3.2)	9.0 (3.0)	16.8 (4.5)	13.4 (5.1)	<0.001*
DISCERN	26.3 (6.6)	25.9 (5.2)	27.3 (5.6)	20.1 (3.0)	28.9 (6.7)	25.5 (5.5)	0.015*
Category	Weighted Kappa			P value			
QUEST	0.742			< 0.001*			
DISCERN	0.569			< 0.001*			

Further analysis utilizing Dunn’s test with Bonferroni correction revealed which social media platforms differed significantly from one another based on QUEST and DISCERN scores. In QUEST, TikTok consistently scored lower than the other platforms. Specifically, Instagram scored significantly higher than TikTok with a mean difference of 6.66 (p < 0.001), while TikTok scored significantly lower than X with a mean difference of -7.83 (p < 0.001). Facebook also scored higher than TikTok (mean difference = 3.91, p = 0.44), but this difference was not statistically significant. No significant differences were observed between YouTube and other platforms. Similar trends were observed for DISCERN scores. TikTok scored significantly lower than X (mean difference = -8.83, p < 0.001), indicating a notable difference. Although Facebook (mean difference = 6.17, p = 0.21) and Instagram (mean difference = 5.83, p = 0.23) also scored higher than TikTok, these differences were not statistically significant. Similarly to QUEST scores, no significant differences were found between YouTube and other platforms (e.g., YouTube vs. Facebook, mean difference = 0.84, p = 1.00) when using DISCERN (Table 2).

**Table 2:** Pairwise comparison of social media platforms for QUEST and DISCERN scores Pairwise comparisons were conducted using Dunn's test with Bonferroni correction to evaluate the differences in mean scores across various social media platforms for both QUEST and DISCERN evaluation instruments. *Denotes statistical significance QUEST: Quality Evaluation Scoring Tool; DISCERN: Discerning the Quality of Written Consumer Health Information

Evaluation Instruments	Comparison (A and B)	Mean Difference (A - B)	Standard Error	Lower 95% CI	Upper 95% CI	p-value
QUEST	Facebook - Instagram	-2.75	1.66	-6.02	0.51	1.00
	Facebook - Threads	-1.48	2.21	-5.81	2.85	1.00
	Facebook - TikTok	3.91	1.37	1.23	6.59	0.44
	Facebook - X	-3.92	1.59	-7.04	-0.80	0.53
	Facebook - YouTube	-0.54	2.01	-4.48	3.40	1.00
	Instagram - Threads	1.28	2.19	-3.01	5.56	1.00
	Instagram - TikTok	6.66	1.33	4.06	9.27	<0.001*
	Instagram - X	-1.17	1.56	-4.23	1.89	1.00
	Instagram - YouTube	2.21	1.98	-1.68	6.10	1.00
	Threads - TikTok	5.38	1.97	1.52	9.24	0.47
	Threads - X	-2.44	2.13	-6.62	1.74	1.00
	Threads - YouTube	0.93	2.46	-3.89	5.75	1.00
	TikTok - X	-7.83	1.24	-10.26	-5.40	<0.001*
	TikTok - YouTube	-4.45	1.74	-7.87	-1.03	0.23
	X - YouTube	3.38	1.93	-0.40	7.15	0.32
DISCERN	Facebook - Instagram	0.34	3.04	-5.63	6.31	1.00
	Facebook - Threads	-1.05	4.08	-9.04	6.95	1.00
	Facebook - TikTok	6.17	2.70	0.88	11.47	0.21
	Facebook - X	-2.66	2.96	-8.47	3.15	1.00
	Facebook - YouTube	0.84	3.05	-5.15	6.82	1.00
	Instagram - Threads	-1.39	3.65	-8.55	5.77	1.00
	Instagram - TikTok	5.83	2.01	1.90	9.77	0.23
	Instagram - X	-3.00	2.35	-7.60	1.60	1.00
	Instagram - YouTube	0.49	2.46	-4.32	5.31	1.00
	Threads - TikTok	7.22	3.38	0.61	13.84	0.35
	Threads - X	-1.61	3.59	-8.64	5.42	1.00
	Threads - YouTube	1.88	3.66	-5.29	9.06	1.00
	TikTok - X	-8.83	1.88	-12.52	-5.14	<0.001*
	TikTok - YouTube	-5.34	2.02	-9.30	-1.38	0.17
	X - YouTube	3.49	2.36	-1.12	8.11	1.00

When analyzing by specific criteria of the QUEST and DISCERN rubric, QUEST scores revealed Instagram exhibited significantly higher authorship scores (mean = 1.39, SD = 0.70) compared to Facebook (mean = 0.57, SD = 0.53) (p = 0.013). X had notably higher attribution scores (mean = 1.72, SD = 0.80) than TikTok (mean = 0.00) (p < 0.001). Complementarity scores showed significant variation, with X (mean = 0.78, SD = 0.31) scoring higher than Facebook (mean = 0.21, SD = 0.39) and TikTok (mean = 0.18, SD = 0.34), with a p-value of <0.001. Tone scores also differed significantly, with YouTube (mean = 1.15) scoring higher than TikTok (mean = 0.30), p < 0.001. For DISCERN scores, significant differences were noted in the clarity of information sources, where X (mean = 3.58, SD = 1.82) scored higher than Facebook (mean = 1.93, SD = 1.54) and TikTok (mean = 1.00, SD = 0.00), (p = 0.003). Similarly, clarity of date information scores were higher for X (mean = 3.17, SD = 1.69) compared to Facebook (mean = 1.86, SD = 1.57) and TikTok (mean = 1.00, SD = 0.00) (p = 0.002) (Table 3).

**Table 3:** QUEST and DISCERN scores by question for each social media site. *Kruskal-Wallis rank sum test QUEST: Quality Evaluation Scoring Tool; DISCERN: Discerning the Quality of Written Consumer Health Information

Characteristic	Facebook (N = 7), mean (SD)	Instagram (N = 9), mean (SD)	Threads (N = 3), mean (SD)	TikTok (N = 20), mean (SD)	X (N = 18), mean (SD)	YouTube (N = 10), mean (SD)	P-value^*^
QUEST:							
Authorship	0.57 (0.53)	1.39 (0.70)	0.67 (0.58)	1.23 (0.53)	1.44 (0.51)	1.25 (0.42)	0.013
Attribution	0.81 (0.96)	0.76 (0.71)	0.89 (0.84)	0.00 (0.00)	1.72 (0.80)	0.62 (1.13)	<0.001
Conflict of Interest	1.57 (0.61)	1.89 (0.22)	1.83 (0.29)	1.55 (0.51)	1.81 (0.49)	1.50 (0.47)	0.11
Currency	1.93 (0.19)	2.00 (0.00)	2.00 (0.00)	2.00 (0.00)	1.72 (0.46)	1.85 (0.34)	0.092
Complementarity	0.21 (0.39)	0.61 (0.33)	0.50 (0.50)	0.18 (0.34)	0.78 (0.31)	0.50 (0.47)	<0.001
Tone	1.00 (0.58)	1.22 (0.51)	1.00 (0.00)	0.30 (0.47)	0.75 (0.62)	1.15 (0.67)	<0.001
DISCERN:							
Clear Aims	4.64 (0.75)	4.67 (0.71)	3.33 (1.04)	4.56 (0.73)	4.47 (0.78)	4.45 (0.83)	0.2
Achieves Aims	4.36 (0.63)	4.06 (0.98)	4.83 (0.29)	3.39 (1.22)	4.11 (0.92)	4.55 (0.83)	0.2
Relevant	4.79 (0.57)	4.67 (0.71)	4.67 (0.29)	4.00 (1.03)	4.75 (0.49)	5.00 (0.00)	0.053
Clear Sources	1.93 (1.54)	2.61 (1.58)	3.33 (2.08)	1.00 (0.00)	3.58 (1.82)	1.60 (1.35)	0.003
Clear Date	1.86 (1.57)	1.67 (1.35)	3.67 (2.31)	1.00 (0.00)	3.17 (1.69)	1.40 (1.26)	0.002
Balanced and Unbiased	4.64 (0.75)	4.56 (0.73)	5.00 (0.00)	3.94 (1.38)	4.58 (0.97)	4.90 (0.21)	0.3
Additional Sources	2.07 (1.37)	1.89 (1.29)	1.50 (0.50)	1.00 (0.00)	2.22 (1.47)	2.10 (1.39)	0.13
Areas of Uncertainty	1.29 (0.76)	1.83 (0.87)	1.00 (0.00)	1.22 (0.67)	2.06 (1.45)	1.45 (1.12)	0.13

## Discussion

Analyzing the quality of healthcare-related information on social media is crucial for physicians, given the prominent use of social media amongst patients as a source of healthcare information. When utilized effectively, social media can serve as a valuable tool for physicians, healthcare organizations, and support groups to provide educational resources and enhance awareness of rare diseases such as BP among different communities. However, incorrect, misleading, and biased information is also prevalent on social media [8]. Our study aimed to analyze the use of social media among patients with BP and to understand the type and quality of health information accessible to them online. 

Our analysis across multiple social media platforms reveals a diverse spectrum of health information regarding BP. Each platform has distinct strengths in engaging audiences and providing educational content. Given the unique characteristics of each social media platform, we found differing QUEST and DISCERN scores underlying the quality and reliability of each platform. An explanation for the differences in QUEST and DISCERN scores could be the type and quality of information presented on each of the social media platforms, reflecting the unique quality type and user demographics of each platform [9].

For instance, X has the highest average scores for both QUEST and DISCERN scores, (16.8 and 28.9, respectively), indicating that content related to BP on this platform may be of higher quality compared to other platforms. One potential reason for the comparatively higher performance of X could be its long-standing adoption by healthcare professionals, specialty organizations, and advocacy groups that share vetted medical information and peer-reviewed research. This professional usage may contribute to a higher proportion of evidence-based content, thereby elevating the QUEST and DISCERN scores of X.

TikTok had the lowest average QUEST score, which might suggest that the content on this platform is not as comprehensive or reliable as other platforms (Table 1). A plausible explanation for this is that TikTok generally attracts a younger demographic of users that often favor more visual and less detailed content to gather information. This preference may explain the platform’s lower average QUEST score than platforms like X, which caters to an older demographic that typically engages more with textual content and less with visual information [10]. YouTube had a DISCERN score of 25.5. These high DISCERN scores suggest YouTube’s effectiveness in delivering detailed and reliable health information through video content, likely due to the platform's capacity for longer, in-depth presentations [11]. Comparatively, platforms like Instagram (QUEST = 15.6, DISCERN = 25.9) and Facebook (QUEST = 12.9, DISCERN = 26.3), characterized by shorter, more visually engaging content, tended to offer a different approach to health education, focusing on disease awareness and community support, despite their varied quality as reflected in their QUEST and DISCERN scores. X (QUEST = 16.8, DISCERN = 28.9) and Threads (QUEST = 14.3, DISCERN = 27.3) stand out in their ability to facilitate rapid information exchange and fostering peer support among patients, providers, and the general public, highlighting their potential for facilitating real-time communication during health-related crises. 

These findings contribute to the broader understanding of social media's role in public health communication. Suarez-Lledo and Alvarez-Galvez, in 2021, identified similar challenges with health misinformation on social media, specifically on platforms that prioritize entertainment over educational content, such as TikTok [8]. Our study supports these findings, showing that despite TikTok's extensive reach, the quality of health information remains substandard compared to platforms like X and YouTube. Additionally, the rapid dissemination of content on social media, driven by engagement algorithms often prioritizes virality over accuracy, further exacerbating the spread of misinformation [4]. This underscores the need for active participation by healthcare professionals to guide and correct public discourse. Smailhodzic et al. (2016) reported that social media could serve as a valuable tool for patient education, if appropriately leveraged [11]. 

Our findings support the use of social media as an essential resource for understanding patient experiences and concerns. Online sources such as message boards and discussion forums provide insight into the common misunderstandings and areas of improvement in disease comprehension and management [12-14]. Moreover, while traditional methods of measuring patient experiences, such as self-reporting and questionnaires, remain important [15], integrating data from platforms like Facebook, X, and Threads could further enhance healthcare quality improvement efforts.

Previous research by Moorhead et al. (2013) emphasized variability in information quality across platforms, with YouTube and X/Twitter providing more reliable content [16]. Our study findings align with this, with X scoring highest on QUEST and DISCERN metrics; however, YouTube did not score as high as expected. Similar studies demonstrate that TikTok has lower reliability, as was found significantly for QUEST and DISCERN in this study, raising concerns about the spread of misinformation among younger audiences who primarily use this platform [8,16]. Chou et al. (2009) noted that interactive platforms such as TikTok often suffer from a lack of authoritative oversight, leading to a higher prevalence of low-quality health information [9]. 

We acknowledge that user demographics, such as age and educational background, can critically influence how health information is created, consumed, and perceived on different platforms. Younger demographics, for example, may favor TikTok, potentially contributing to the lower reliability scores we observed. Future studies should incorporate demographic analyses to better understand how engagement and credibility perceptions vary among user groups.

Future research should focus on improving the content quality on lower-performing platforms. Expanding the validation of tools like QUEST and DISCERN and developing platform-specific guidelines could enhance the reliability and educational value of social media content [6,7]. Artificial intelligence (AI)-driven content verification systems could also be integrated into social media platforms to flag or demote low-quality health information, based on such guidelines [12]. Additionally, future investigations would benefit from qualitative content analyses to identify recurring themes of misinformation and understand their potential impact on patient decision-making. Such analyses could provide deeper insights into how misinformation evolves and how targeted educational strategies might mitigate its influence. Longitudinal studies should explore the impact of such intervention on public health outcomes, particularly for emerging health crises where misinformation can have serious consequences.

Limitations

This study has several limitations. This cross-sectional analysis should only be considered a snapshot of social media content as of 2024. Additionally, our study is limited to a convenient sample of six platforms and posts highly related to BP, so our sample excludes other posts and platforms that may also play a supplementary role in social media medical content. This study furthermore does not account for the variability in search results for different users based on each social media platform’s algorithm, and also did not account for temporal variability in algorithmic search results, as all searches were completed on the same day.

While our study highlights the potential of social media in enhancing public health literacy on rare diseases, it also underscores significant limitations, particularly concerning content quality and audience reach. The varying QUEST and DISCERN scores across platforms hint at an inconsistency in the reliability and depth of health information, echoing concerns about misinformation and the need for critical evaluation by users [4,16].

Moreover, our analysis is limited by its focus on English-language content, potentially overlooking how social media is used for health communication in different linguistic, cultural, and socioeconomic settings. Future research should strive for a more global perspective, examining the role of social media in health education across varied socioeconomic and demographic landscapes, and evaluating strategies to mitigate misinformation and improve the quality and reach of health information.

## Conclusions

Social media continues to revolutionize the way organizations, physicians and everyday people connect to communicate health-related information. Healthcare professionals must understand the educational quality of social media content surrounding the topic of diseases, like BP, to improve awareness and patients’ perceptions of their disorder through their interactions on social media. We underscore the importance of ongoing research in this field. Given the dynamic nature of social media and its impact on public health literacy, we advocate for longitudinal studies to monitor changes in the quality of information and user engagement over time. Additionally, intervention studies designed to enhance the accuracy and helpfulness of health-related content could significantly benefit public health outcomes.
